# Purification and Characterization of NDH-2 Protein and Elucidating Its Role in Extracellular Electron Transport and Bioelectrogenic Activity

**DOI:** 10.3389/fmicb.2019.00880

**Published:** 2019-05-07

**Authors:** K. Vamshi Krishna, S. Venkata Mohan

**Affiliations:** Bioengineering and Environmental Sciences Laboratory, EEFF Centre, CSIR-Indian Institute of Chemical Technology, Hyderabad, India

**Keywords:** NADH dehydrogenase II, electron flux, extracellular electron transport, bioelectricity, bioelectrochemical systems

## Abstract

In microbial electrochemical systems, transport of electrons from bacteria to an electrode is the key to its functioning. However, the roles of several electron transport proteins, especially the membrane-bound dehydrogenases which link cellular metabolism to EET pathway are yet to be identified. NDH-2 is a non-proton pumping NADH dehydrogenase located in the inner membrane of several bacteria like *Bacillus subtilis*, *Escherichia coli*, etc. Unlike NADH dehydrogenase I, NDH-2 is not impeded by a high proton motive force thus helping in the increase of metabolic flux and carbon utilization. In the current study, NADH dehydrogenase II protein (NDH-2) was heterologously expressed from *B. subtilis* into *E. coli* BL21 (DE3) for enhancing electron flux through EET pathway and to understand its role in bioelectrogenesis. We found that *E. coli* expressing NDH-2 has increased the electron flux through EET and has shown a ninefold increase in current (4.7 μA) production when compared to wild strain with empty vector (0.52 μA). Furthermore, expression of NDH-2 also resulted in increased biofilm formation which can be corroborated with the decrease in charge transfer resistance of NDH-2 strain and increased NADH oxidation. It was also found that NDH-2 strain can reduce ferric citrate at a higher rate than wild type strain suggesting increased electron flux through electron transport chain due to NADH dehydrogenase II activity. Purified NDH-2 was found to be ∼42 kDa and has FAD as a cofactor. This work demonstrates that the primary dehydrogenases like NADH dehydrogenases can be overexpressed to increase the electron flux in EET pathway which can further enhance the microbial fuel cells performance.

## Introduction

Bioelectrochemical systems have made the possibility of treating organic pollutants in wastewaters with concurrent generation of energy in the form of bioelectricity into reality. They use electroactive bacteria (EAB) as biocatalysts and their electrode interactions to drive electrons from oxidation of organic compounds for bioelectricity generation (Logan, 2009; Venkata Mohan et al., 2014; Santoro et al., 2017). To date, a wide range of EAB were identified that exchange electrons with insoluble electron acceptors either directly or indirectly (Lovley et al., 2011; Sydow et al., 2014; Kracke et al., 2015). Indeed, the electron transfer mechanism of each species varies significantly from the other such that there are several mechanisms for direct electron transfer, mediated electron transport and other modes of extracellular electron transport depending on the organism. However, there are several bottlenecks in the up scaling of bioelectrochemical systems; especially due to the low rate of extracellular electron transfer. Recent years have seen several studies focused on genetic engineering approaches to optimize the rate of extracellular electron transfer and enhancement of electroactive biofilm formation to obtain high power yields (Venkata Mohan et al., 2009; Jensen et al., 2010; Han et al., 2016).

Several studies with *Bacillus subtilis* have shown that the strain is capable of extracellular electron transport via mediated electron transport and was shown to generate significant voltages in both dual and single chamber configurations (Coman et al., 2009; Nimje et al., 2009). It was also found to be part of several electroactive biofilms operated with mixed culture inoculums (Vamshi Krishna and Venkata Mohan, 2016). The fundamental molecular mechanism of EET of *B. subtilis* is not entirely understood till now. However, type II NADH:quinone oxidoreductase or NADH dehydrogenase II was found to be major NADH dehydrogenase in *B. subtilis* which regulates the NADH/NAD+ ratio in the cytoplasm and thereby the metabolic state of the cell (Gyan et al., 2006). NDH-2 is a non-proton pumping membrane-bound protein involved in the respiratory pathway of many bacteria and is responsible for NAD^+^ regeneration through NADH dehydrogenase activity (Marreiros et al., 2016). The structure of NDH-2 is relatively small and is approximately 42 kDa and bound non-covalently with a single flavin adenine dinucleotide (FAD) as the only coenzyme molecule (Kerscher, 2000).

One significant property associated with NDH-2 is its ability to uncouple NADH oxidation and proton transport across the membrane which can otherwise lead to the development of a high proton motive force that can, in turn, impede the respiratory process as in case of NDH-1. Expression of NDH-2 can lead to higher metabolic rates and especially when there is high energy available to the cell (Gyan et al., 2006). It is well understood from the earlier studies that an appropriate ratio of electron carriers like NAD can play an important role in BES (Han et al., 2016). The NAD pool (both oxidized and reduced forms) majorly represents the intracellular pool of electrons for respiratory electron transport chain and EET (Berríos-Rivera et al., 2002b; Reddy et al., 2010). Previous studies on NAD have focused on the either increasing the NADH or total NAD pool by the expression of enzymes like formate dehydrogenase (Han et al., 2016) or by using systems biology approach (Li et al., 2018) which can in turn influence electricity generation in MFCs. In this study we focused on the improvement of the bioelectrochemical performance of BES through genetic engineering of *Escherichia coli* cells which can increase the rate of NADH oxidation there by increasing the electron flux into the electron transport chain and EET. The results obtained have shown that expression of *ndh* gene successfully increased ratio of NAD^+^/NADH and total NAD pool in the cells which in turn increased the bioelectricity production.

## Materials and Methods

### Strains and Growth Conditions

All strains, unless otherwise specified, were grown in Luria bertani (LB) media at 37°C with 50 μg/mL kanamycin and preserved as glycerol stocks at −80°C. Glycerol stocks were used to inoculate 5 mL of LB broth, and cultures were grown overnight at 37°C with 250 rpm shaking. Then overnight cultures were back-diluted into LB media at a dilution of 1:100 and grown with 250 rpm shaking till an OD of 0.8 is reached. Expression of the *ndh* gene was induced by addition of isopropyl β-D-1-thiogalactopyranoside (IPTG) at a concentration of 1 mM to the media. Cells were further grown for 3 h before being harvesting. For bioelectrochemical system operation and iron reduction assays, induced cells were washed with M9 minimal salts solution, and grown in M9 minimal media supplied with wolfe’s vitamins, minerals, and appropriate antibiotics, as necessary.

### Cloning and Expression of NDH-2

The *ndh* gene (BSU12290 or NCBI gene ID: 939832) of *B. subtilis* (Genbank accession number: KX470414.1) that has been isolated from the long term operated BES reactor was amplified using synthetically designed primers (Table 1) with BamH1 and HindIII enzyme restriction sites at the downstream of start and stop codons. The amplicon with the desired gene sequence and pET-28a(+) (Novagen) with a polyhistidine-tag at its N-terminal were subjected to restriction digestion with high fidelity BamH1 and HindIII (NEB, United States) restriction enzymes at 37°C for overnight followed by ligation with T4 DNA ligase (NEB, United States) at 16°C overnight. Further, recombinant vector with *ndh* gene is transformed into the *E. coli* BL21 (DE3) cells for protein expression and stored as glycerol stocks at −80°C. The transformed cells were then used for expression of *ndh* gene in two stages according to the protocol described above. The induced culture was further incubated to 3 h and then harvested in centrifuge at 4°C (8000 rpm/5 min). Cell pellet was further processed based on downstream application.

**Table 1:** List of primers used in the study.

For cloning and expression of *ndh* gene		
Gene	Enzyme	Primer sequence (5′ to 3′)
***ndh***	NADH dehydrogenase II	GCAGGAGGATCCATGTCAAAACATATTGTC
		ATATGAAAGCTTAGGTAAGCCAGGCTGAA

### Protein Purification

The cells lysis of 2 liter culture, grown and harvested as above was carried out by resuspending the cell pellet in 100 ml of lysis buffer (50 mM Tris, 500 mM NaCl, 5% glycerol, 2 mM pefabloc, 2 μl benzonase) followed by incubation at room temperature for 30 min. Further, the cells were sonicated with a pulse rate of 2 s on and 3 s off cycle (50% amplitude) for 5 min on ice, after which the lysate is centrifuged at low temperature (13,000 rpm/30 min) to remove cell debris. The His-tagged membrane bound NDH-2 was further purified through affinity chromatography by making use of 5 ml of Ni-NTA beads (GE, Healthcare). The supernatant was incubated with the equilibrated resin at 4°C for 1 h to enhance the binding of NDH-2 and is then transferred to a gravity flow column. The unbound proteins in the column were washed with 20 column volumes of wash buffer (50 mM Tris, 500 mM NaCl, 5% glycerol), after which the NDH-2 bound with resin was eluted with a gradient of 5–100% of immidazole (500 mM imidazole = 100%) in the above buffer and collected in multiple fractions. The column purified protein is then dialyzed to remove imidazole. The fractions collected during the chromatography were loaded on to the SDS-PAGE gel (12% separating gel) to check for the purity. Further, the protein confirmation was done through western blotting by transfer of proteins separated during the electrophoresis onto the PVDF membrane through electro-blotting (25 V) for overnight at low temperature. Later, the membrane was blocked with 2% of blocking agent and then incubated at 4°C with mouse anti-his primary antibodies, followed by incubation with HRP linked secondary antibody, and visualized through luminal based detection.

### Bioelectrochemical System Design and Characterization

The harvested cells were washed thrice with M9 minimal media before their use in BES and resuspended in the same, supplemented with wolfe’s trace metals, vitamins and 20 mM lactate as a carbon source. Cells were adjusted to OD of 1 and purged with O_2_ free N_2_:CO_2_ (80:20) mixture prior to its inoculation into BES. The role of NDH-2 in EET was evaluated using single chambered BES with a working volume of 60 ml using FTO plate anode with a surface area of 6 cm^2^, platinum wire as cathode and Ag/AgCl (Sat’d KCl) as a reference electrode. For Chronoamperometry study, Toray carbon paper was used as anode, graphite rod as cathode and Ag/AgCl (Sat’d M KCl) as a reference electrode. Culture preparation and reactor setup was carried out in the anaerobic glove box (Coy Laboratories) supplied with oxygen free mixture gas containing nitrogen (85%), carbon di oxide (10%), and hydrogen (5%). Protein film voltammetry was performed using thin layer quartz glass spectroelectrochemical cell Kit (ALS) using platinum mesh working electrode and platinum wire counter electrode with Ag/AgCl (Sat’d KCl) as reference electrode.

### Bioelectrochemical Analysis

To the anode of BES, −160 mV of potential was applied using multi-channel potentiostat-galvanostat system (Biologic VMP-3, France) to enhance the bacterial growth on the anode (biofilm formation) and then the current generated was measured. Riboflavin at a final concentration of 5 μM was added to the reactor for mediator based studies. Cyclic Voltammograms were recorded for both induced and wild-BES at 1 mV/s scan rate in the scan range of +0.5 to −0.5 V using VMP-3 potentistat (Raghavulu et al., 2012). For protein voltammetry, voltammograms were detected at a scan rate on 10 mV/s in the range of +1 to −1 V. To understand the growth of biofilm on the electrode, potentio electrochemical impedance spectroscopy (PEIS) was performed in a frequency range of 100 MHz to 10 mHz and a potential of + 200 mV was applied on to the anode during the recording of the technique. All the potentials discussed in the current work are with respect to the Ag/AgCl, KCl (Sat’d KCl) reference electrode unless stated otherwise.

### Estimation of Biofilm Using Field Emission Scanning Electron Microscope (FE-SEM)

The biofilm formation on the anode after the BES operation (chronoamperometry) was evaluated for respective reactors using FE-SEM. The anodes from both the reactors were removed as soon as the reactor was opened and washed with phosphate buffered saline to remove the unbound and loosely bound cells. The biofilm was then fixed in 3% glutaraldehyde in phosphate buffered saline for overnight at 4°C. After fixation, electrodes were washed twice in PBS and sequentially dehydrated using increasing concentrations of ethanol (10–100% for 10 min at each concentration). After two final changes in 100% in ethanol, cells were desiccated overnight in vacuum desiccator and processed immediately for SEM by sputter coating the evaporated silver on the biofilm at a thickness of 10 nm and viewed using Apreo LoVac electron microscope (Field Electron and Ion) at an operating voltage of 20 kV.

### NAD/NADH Estimation

The ratio of NAD/NADH in both induced and wild type cultures was estimated using NAD/NADH Quantitation Kit (Sigma Aldrich). The harvested cells from both induced and wild type cultures were washed thoroughly with chilled phosphate buffered saline and extraction of NADH/NAD was performed in NADH extraction buffer by 2 freeze thaw cycles in liquid nitrogen for 20 min (10 min in Liquid N_2_ and 10 min at room temperature). Debris was removed by centrifugation at 13,000 g for 10 min and the resulting supernatant containing NAD/NADH was deprotinized by passing it through 10 kDa protein cutoff spin filter to remove NADH consuming proteins. Deprotinized supernatant was used in a NAD cycling assay to determine the amounts of NADH and total NAD content following the manufacturer’s instructions. The colorimetric estimation of the NADH in the sample along with standards was done using Multimode plate reader (SpectraMax M2e, Molecular Devices) at a wavelength of 450 nm. All the steps were carried out at low temperature in order to maintain stability of NADH.

### Iron Utilization Assays Using Ferrozine Assay

The role of NDH-2 in extracellular electron transport was evaluated through iron reduction assay (ferrozine assay). Initially the cells were grown in LB broth, following the induction of NDH-2, cells from both induced and wild type cultures were pelleted, and washed with M9 minimal media. Washed pellet was resuspended in M9 minimal media separately with lactate as electron donor and ferric citrate (10 mM) as electron acceptor at an OD of one in anaerobic glove box (Coy, AR, United States). The kinetics of iron reduction [Fe (II)] in both the cultures was studied by Ferrozine assay (Ruebush et al., 2006).

## Results and Discussion

### Protein Purification

**FIGURE 1:**
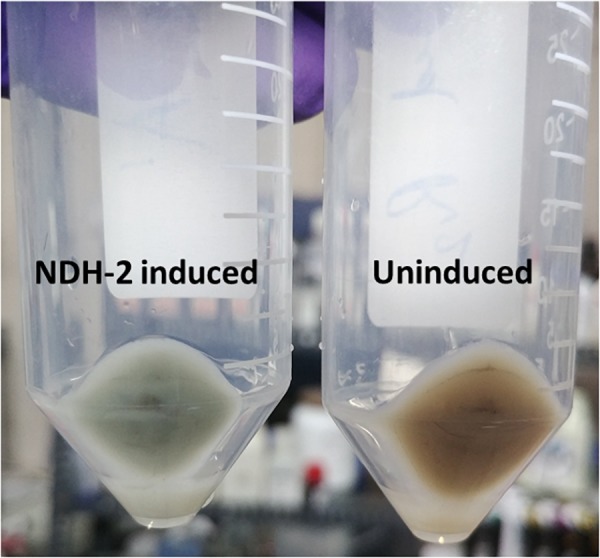
Cell pellets of NDH-2 induced (1 mM IPTG) and uninduced cultures, Green color of the pellet is due to the presence of FAD cofactor in the NDH-2 enzyme.

NADH dehydrogenase II protein is non-proton pumping type II NADH dehydrogenase which plays a central role in the respiratory metabolism of bacteria. Its ability to overcome the inhibition posed by high proton motive force makes it an ideal protein to study for increasing the metabolic and electron flux through the cellular metabolism and electron transport chain. The gene encoding NDH-2 was transformed into *E. coli* BL21 (DE3) using pET28a vector. The insertion of *ndh* gene into the vector was confirmed by double digestion using BamHI and HinDIII restriction enzymes, while the gene orientation of *ndh* was confirmed by the sequencing of the recombinant vector. Cells expressing NDH-2 protein were light greenish to yellowish in color compared to the cream color of wild type pellet (Figure 1) indicating the expression of the NDH-2 protein. Green color of the protein was due to the presence of non-covalently bound FAD cofactor in the NDH-2 protein (Heikal et al., 2014).

**FIGURE 2:**
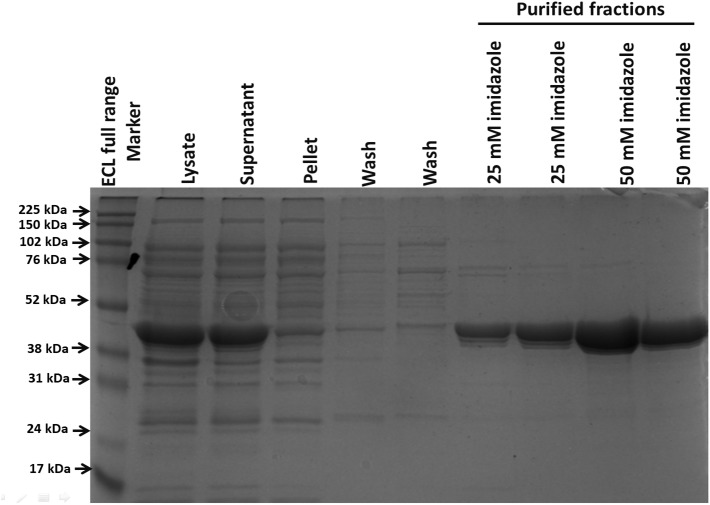
Protein purification using his tag purification (Ni-NTA beads). Purified protein was light greenish in color emphasizing the presence of FAD^+^ cofactor in the NDH-2.

**FIGURE 3:**
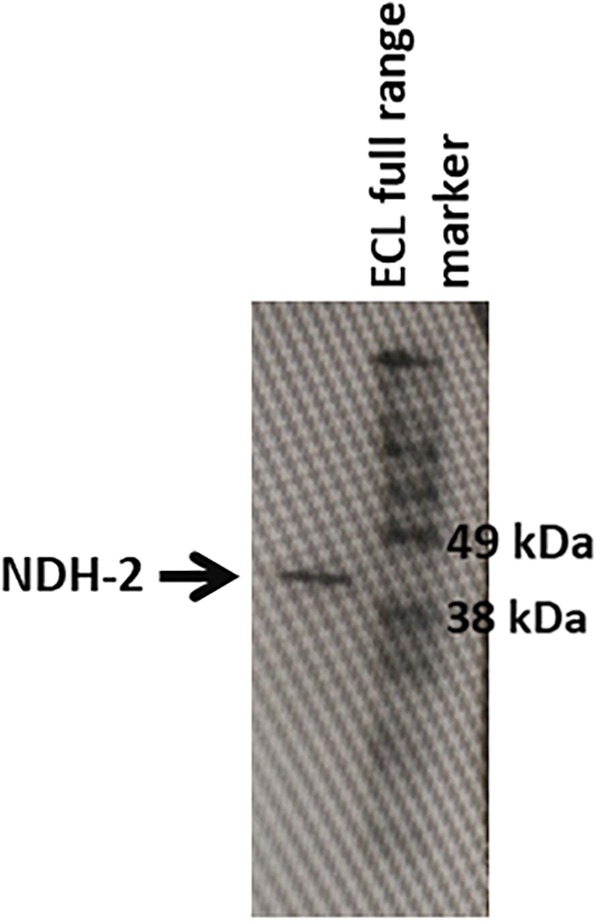
Western blotting of the NDH-2 protein using anti-6X his antibodies. Prescence of band indicates the prescence of the NDH-2 in the membrane fraction.

**FIGURE 4:**
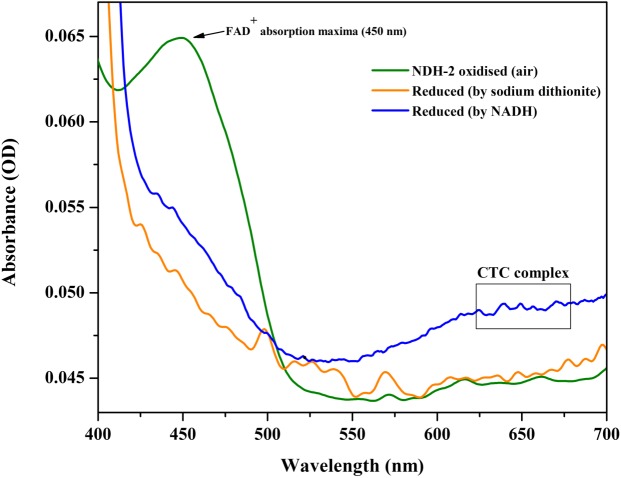
Absorption (visible) spectra of purified NDH-2 under oxidizing (air) and under reducing condition (reduced by NADH and sodium dithionite). Addition of dithionite has resulted in reduction of FAD+ which lead to the overall decrease in absorbance at 450 nm. While NADH reduction has resulted in formation of CTC along with diminishing of absorbance maxima at 450 nm.

After its successful expression in *E. coli* BL21 cells, the His-tagged NDH-2 was then purified from total protein fraction by Ni-NTA affinity chromatography using his-tag of NDH-2. Purity of the protein was checked on 12% SDS PAGE which showed good quantity of purified protein near 42 kDa (Figure 2). As the localization of heterologously expressed NDH-2 is important, western blotting with an anti his antibody has been performed which has produced a band at the expected molecular mass of NDH-2, i.e., nearly 42 kDa, indicating proper expression of protein (Figure 3). Redox activity of the NDH-2 was probed by using UV-Vis spectroscopy. Visible spectra of oxidized NDH-2 have showed a sharp peak at 450 nm indicating the presence of FAD^+^ cofactor in the enzyme which has an absorption maxima at 450 nm in its oxidized form. (Salewski et al., 2016). Reduction of NDH-2 using sodium dithionite and NADH has resulted in gradual decrease in 450 nm peak which finally diminished indicating the reduction of FAD^+^ to FADH_2_ by sodium dithionite and NADH which has no absorbance at 450 nm. In case of NADH reduction, a charge transfer complex or CTC (intermediate enzyme, NAD complex) was observed at ∼650 nm (Figure 4). While in case of sodium dithionite, no CTC formation was observed indicating that it has the ability to reduce FAD^+^ but lacks the ability to form CTC which is essential for the enzyme activity (Blaza et al., 2017).

### Bioelectrogenesis of NDH-2 Clone

NADH dehydrogenase II induced cells after transferring into reactor, were purged with oxygen free nitrogen, carbon dioxide mixture (80%: 20%, V/V) for half an hour to remove any traces of oxygen. Chronoamperometry was not performed during the first hour for acclimatization, as cells would take time to synthesize the machinery required for anaerobic growth and lactate utilization. Current producing ability of the NDH-2 induced cells was compared with wild type cells where NDH-2 overexpression was absent. An applied potential of −0.160 V was continuously applied on the anode of both the systems to promote the growth of bacteria (biofilm formation) (Liu et al., 2008).

#### Chronoamperometry (CA)

**FIGURE 5:**
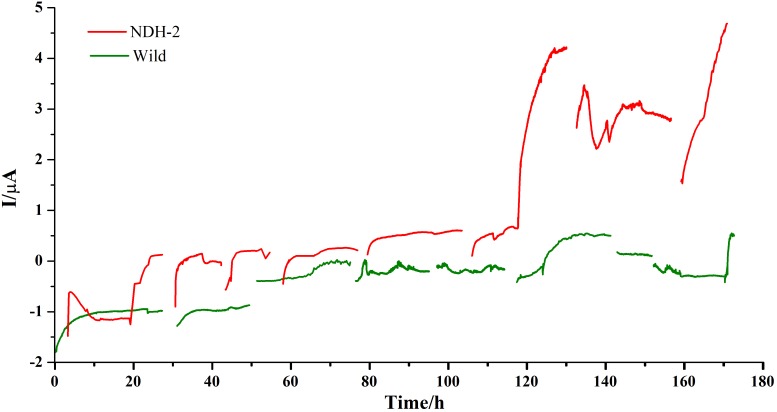
Bioelectrogenic activity of NDH-2 and wild BES reactors poised at – 0.160 V. NDH-2 induced culture has shown ninefold increase in current production compared to uninduced BES reactor.

Chronoamperometry (CA) was performed at −160 mV for 7 days (each cycle comprising of 24 h) to promote the biofilm formation and also to enhance the extracellular electron transport. A potential of −160 mV vs. Ag/AgCl (Sat’d KCl) was chosen to apply on anode in order to facilitate the biofilm formation on anode and also to increase the interaction of quinone pool with electrode. In *E. coli* and several other bacteria, dimethyl menaquinone (DMK) was found to be predominant quinone shuttling the electrons between primary hydrogenases and the final electron acceptors. DMK has a midpoint potential of ∼+40 mV vs. SHE or ∼−156 mV vs. Ag/AgCl (Sat’d KCl). Therefore it is hypothesized that application of potential near to −160 mV can result in transport of electrons from quinone cycle to the electrode. A blank reactor with uninoculated media and bare electrodes was run to see if any background currents were produced due to the media components and it was found that very low back ground current was produced from the reactor (Supplementary Figure 1A) indicating that the current produced from the reactor was only because of electrogenic activity of the biocatalyst. In both the cases current production was stabilized after ∼5 h of starting the cycle. During the initial cycle, the NDH-BES reactor (+0.12 μA) showed a good increment in current production compared to the wild-BES reactor (−0.9 μA) which is almost 1.5 fold higher (Figure 5). In case of NDH-2 BES, maximum current produced has increased till 3rd cycle (+0.13 μA) and stabilized at 4th cycle (+0.16 μA) which can be observed from steady current production indicating a stable biofilm formation. Whereas, current production from wild BES started increasing only after 2nd cycle (−1.2 μA) of Chronoamperometry and stabilized after 3rd cycle (−0.1 μA). Once the stable biofilm formation was observed in both the reactors (after 4th cycle), riboflavin was injected into both the reactors at a concentration of 5 μM to see its effect on the extracellular electron transport (5th cycle). Riboflavin addition has resulted in increase of current production but with distinct differences between NDH and wild-BES. In NDH-2 BES, current production has started immediately and up surged to the highest value of +4.25 μA by the end of the cycle. In case of wild-BES, current production has increased gradually unlike in NDH-BES and reached its highest point of +0.52 μA by the end of the 5th cycle. Addition of riboflavin has resulted in ninefold increase in current in NDH-BES. To confirm the involvement of riboflavin on the extracellular electron transport, next cycle was operated with fresh media without riboflavin (cycle 6), which has resulted in a decrease of current produced by both the reactors. Addition of riboflavin in the next cycle (7th cycle) has shown performance comparable to the 5th cycle of the respective BES reactors. The results are consistent with the hypothesis that the electrons accumulated at the quinone cycle due to increased NADH oxidation (because to NDH-2 activity) can be shuttled to anode by the addition of mediators preferably flavins which can either interact with the quinone cycle (dimethyl menaquinone; DMK) or DMK reductase. Immediate increase in current production after the addition of riboflavin shows that it was able to tap the electrons in the *E. coli* transport chain and involvement of riboflavin in shuttling the electron from biofilm to the electrode. A nine-fold increase in current production by NDH-BES compared to wild-BES after riboflavin addition is also a proof that the NDH-2 expression has increased the electron flux into the electron transport chain and subsequently into the extracellular electron transport compared to the wild type cells. Missing data in the CA curves were the result of feed or media changes where current was not recorded simultaneously. FE-SEM images were recorded after the completion of the Chronoamperometry cycles.

#### Cyclic Voltammetry (CV)

**FIGURE 6:**
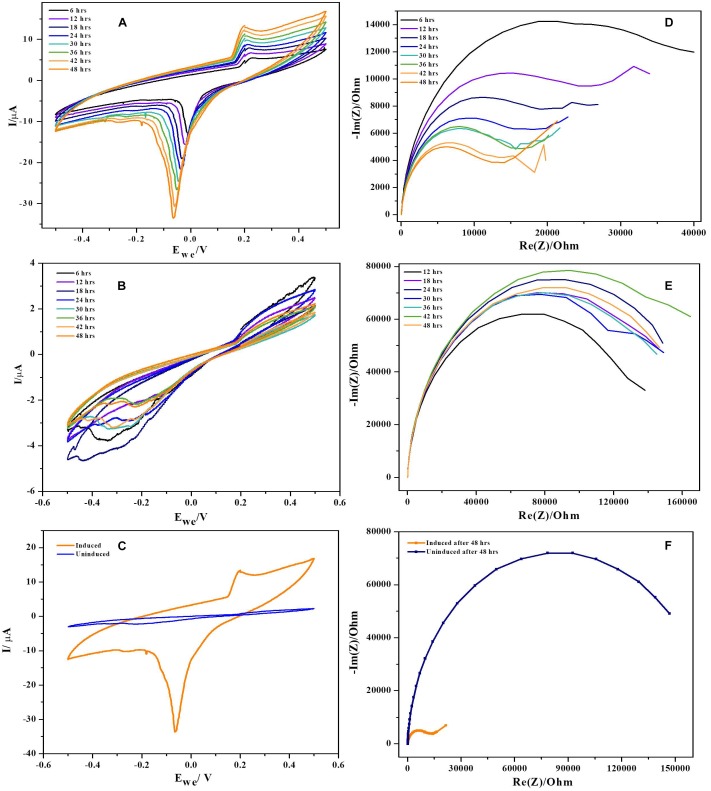
Cyclic voltammograms recorded during 48 h period of time (at every 6 h) at a scan rate of 0.001 Vs^−1^. **(A)** NDH-2, **(B)** wild, and **(C)** combined voltammograms. In case of NDH-2 induced, *E^0‘^* is shifted significantly negative while uninduced has no peaks produced. Biofilm monitoring by impedance spectroscopy, **(D)** NDH-2, **(E)** wild, and **(F)** combined.

Cyclic voltammetry was performed using three electrode configuration at low scan rates (1 mV. S^−1^). To rule out the non-involvement of media components, voltammograms with uninoculated anode and fresh media were generated (Supplementary Figure 1C). It was observed that, the uninoculated BES produced no catalytic current ruling out involvement of media components and exogenous mediators in current generation. CV’s reveal microbial catalyzed redox reactions during substrate oxidation at the anode of BES and also redox reaction kinetics at bacteria-electrode interface. Cyclic voltammograms of NDH-BES and wild-BES at various time points during operation along with comparison was presented in the Figure 6A–C. The cyclic voltammograms of NDH-BES showed both oxidation (*E*_1_) and reduction reaction (*E*_2_) with maximum peak current in the reverse scan. Reduction peak (*E*_2_) with a midpoint potential of ∼−10 mV was observed at the 6th hour of operation which slowly shifted (peak splitting) towards reduction side to ∼−70 mV by the end of 48th hour (Fu et al., 2013). The presence of redox peaks in voltammograms indicates either the involvement of redox mediators or surface bound proteins or cytochromes. When CVs were performed with spent media and fresh electrodes no redox peaks were found indicating that no mediators were involved in the reaction. This suggests the involvement of an unknown surface bound protein or a cytochrome which might have expressed in NDH-2 cells due to the increase in NADH oxidation along with the resulting change in cellular redox balance. However, no considerable peaks were observed with wild-BES neither in forward nor in the reverse scan. Though the redox currents were seen in the wild BES voltammograms, the redox peaks observed in the NDH-BES were not found suggesting that the unknown surface bound protein or cytochrome involved in NDH-BES was not expressed in wild type cells. Though peak shifts were observed in previous *Geobacter* biofilm studies, they were mostly due to the diffusion limited conditions prevailing at thin biofilms due to the increasing scan rate where the diffusion of a redox molecule to the electrode is not as fast as the electrode reduction which results in the peak shift (Richter et al., 2009). But in this case, the peak shift of *E*_2_ was observed with respect to time at same scan rate of 1 mV. S^−1^ indicating that the peak shift generated was not as a result of the diffusion limited current.

The plausible explanation for shift in formal potential of *E*_2_ peak is the biofilm formation on electrode, resulting in various micro-environments which can influence the formal potential of the redox molecule as the same redox mediator of the electron transport chain will be exposed to different environments resulting in peak shift (Chen et al., 2002). Another explanation for the peak shift seen in NDH-2 CV’s can be due to high concentration of redox molecules or electron donor at thin biofilm resulting in lateral electron transfer to the adjacent redox mediators parallel to the surface of the electrode (Strycharz et al., 2011). In this case, it can be understood that, NDH-2 expression has resulted in increased biofilm formation (discussed in the next sections) which has led to the formation of a thin biofilm and one or both of the above discussed mechanisms must have played role in shift of peak potentials.

**FIGURE 7:**
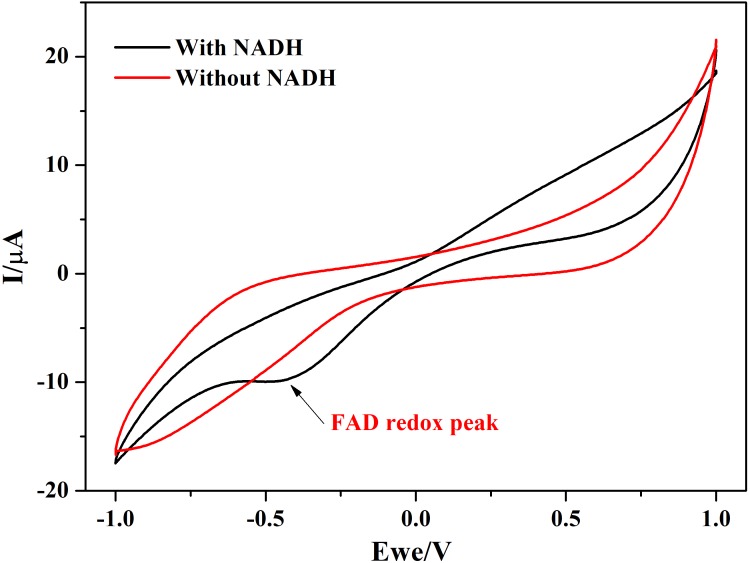
Protein film voltammetry of purified NDH-2 has shown a redox peak at ∼ –225 mV indicating the involvement of FAD cofactor in the enzyme electrode reactions which is also the cofactor present in the enzyme.

In addition to this, protein film voltammograms of purified NDH-2 has shown a characteristic peak at −225 ± 10 mV vs. SHE when the enzyme was reduced with NADH which was absent when no electron donor was added to the reaction (Figure 7). The peak observed in the former case can be attributed to the involvement of the flavin cofactors like FAD which has standard redox potential of −220 mV vs. SHE. It can be clearly seen from the literature and spectral studies (Figure 4) that the cofactor present in NDH-2 is FAD and a peak at −225 mV in voltammograms suggest that the FAD is catalyzing the transfer of electrons from NADH to the electrode or any other electron acceptor like quinones.

#### Potentiostatic Electrochemical Impedance Spectroscopy (PEIS)

Potentiostatic electrochemical impedance spectroscopy was used to understand the biofilm formation and resulting changes in the charge transfer resistance (R_CT_) to transport electrons from biofilm to the anode (Stöckl et al., 2016). Impedance of BES reactor with only media and bare electrodes has shown huge resistance of ∼ 200 kΩ indicating that media components have no role in decreasing the overall resistance of the cell (Supplementary Figure 1B). Results have demonstrated that the charge transfer resistance has decreased over time of operation indicating the deposition of bacteria (biofilm formation) (Ramasamy et al., 2008; Ben-Yoav et al., 2011; Velvizhi and Venkata Mohan, 2012). NDH-BES has showed a six-fold decrement during 48 h of operation. R_CT_ values decreased over time from ∼ 40 kΩ at 6th hour of the experiment to the ∼ 14 kΩ by the end of the cycle. EIS plots of NDH-2 induced at various time points were presented in Nyquist diagrams (Figure 6D–F). The decrease in impedance with time can be correlated to the formation of electroactive biofilm on anode which can reduce the activation losses (transfer of electrons from bacteria to the electrode) (Ben-Yoav et al., 2011). With the formation of biofilm on anode, the resistance for electron transport will decrease as direct electron transport will dominate the mode of extracellular electron transport in BES. While in case of wild-BES, a different pattern of impedance plots were observed with respect to time of operation. R_CT_ values of wild-BES nyquist plot suggest that the charge transfer resistance has increased initially and stabilized over time. At 12th hour of operation, R_CT_ has increased from ∼138 kΩ to ∼148 kΩ, where it got stabilized till 36th hour of operation. Though there was increase in R_CT_ at 42th hour (∼ 162 kΩ), it finally returned to the previous stabilized state of ∼145 kΩ suggesting a lower rate of biofilm formation in wild-BES reactor compared to the NDH-BES.

**FIGURE 8:**
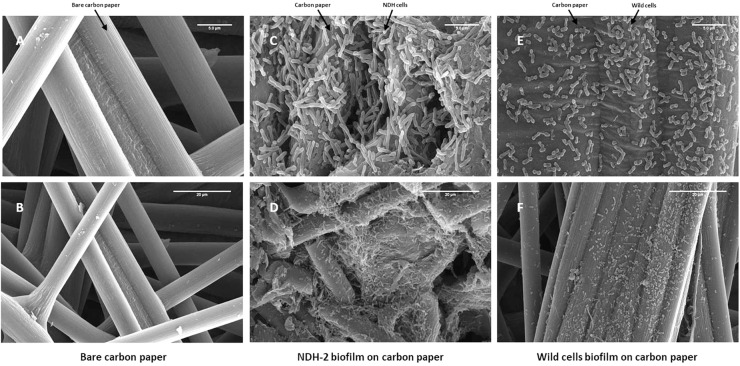
Biofilm formation on the FTO plates after chronoamperometry. Biofilm was captured by using FE-SEM at 20 kV. After seven cycles of applied potential, it was observed that the cell density was more on the NDH-2 BES anode. **(A)** Bare carbon paper at 5 μm scale, **(B)** Bare carbon paper at 20 μm scale, **(C)** NDH-2 induced biofilm at 5 μm scale, **(D)** NDH-2 induced biofilm at 20 μm scale, **(E)** Wild cells biofilm at 5 μm scale, and **(F)** Wild cells biofilm at 20 μm scale.

In addition to the EIS measurements, enhanced formation of biofilm in case of NDH-BES (compared to wild) was also confirmed by biofilm images taken at the end of the chronoamperometry using field emission scanning electron microscopy. Representative image of bare electrode surface was shown in Figure 8. It can be observed that the biofilm formed by NDH-2 cells after 7 days of chronoamperometry was thicker and denser when compared to wild type biofilm. Moreover the biofilm of NDH-BES biofilm was found to be healthier when compared to wild-BES biofilm. Increased biofilm formation in NDH-2 cultures can be because of the excessive electron and metabolic flux generated by the NDH-2 cells due to the activity of NADH dehydrogenase 2. The excessive electrons generated might have favored the NDH-2 cells growth on anode to enhance direct electron transport enabling faster discharge of electrons from bacteria to anode.

### Effect of NDH-2 on NAD/NADH Concentration, Bioelectricity, and Biofilm Development

**FIGURE 9:**
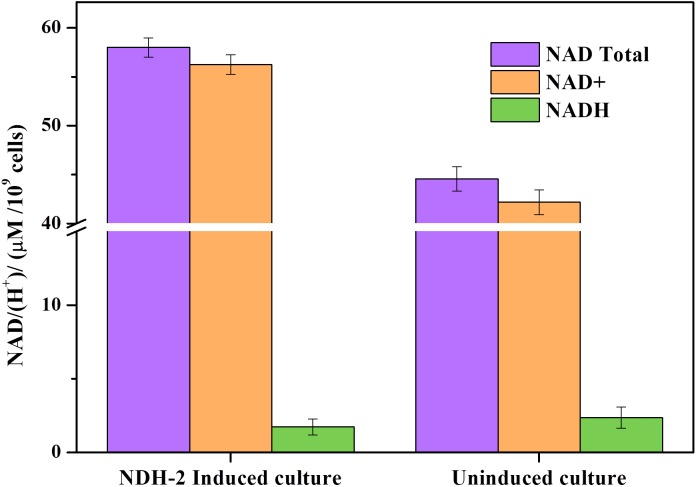
Total NAD^+^, NAD^+^, and NADH concentration of aerobically grown cultures after 3 h of NDH-2 induction. NADH/H^+^ concentrations were found to be very low corresponding to the high rate of catabolism and increased electron flux into electron transport chain.

Expression of *ndh* gene in *E. coli* has resulted in 1.32 fold increase (∼58.68 μM/10^9^ cells) in total NAD (H) pool compared to the wild type culture (∼44.43 μM/10^9^ cells) (Figure 9). In NDH-2 induced cultures NAD^+^ concentration was much higher than the NADH (<2 μM/10^9^ cells) indicating high catabolic rate as a result of NDH-2 activity in the cellular membrane (Berríos-Rivera et al., 2002a). Though the concentration of NADH in wild type cells was negligible (<2 μM/10^9^ cells) it was higher than the induced cells signifying the activity of NDH-2. It can be clearly seen that the bacteria has increased the production of total NAD/(H^+^) to compensate for increased oxidation of NADH by NDH-2 enzyme.

In addition to this, it has also been discovered recently that the enzyme NADH oxidase which catalyzes NAD^+^ regeneration from NADH for maintaining glycolysis has a role to play in biofilm formation in *Streptococcus sanguinis* which suggests that the NAD^+^ regeneration is one of the key factors in biofilm formation (Ge et al., 2016). NDH-2 being an enzyme which oxidizes NADH to NAD^+^ might also play a similar role in biofilm formation as both the enzymes does equivalent function but in different perspectives. From the results it can be understood that intracellular NAD^+^ concentration has substantially increased in NDH-2 induced cells which might have triggered higher biofilm formation (Figure 8). The increased NADH oxidation has increased electrons released to EET pathway and played an important role in bioelectricity generation and overall electron flux through electron transport chain.

In addition to this, positive effect of biofilm formation on the anode can be correlated with electrochemical impedance spectroscopy (EIS) results where it can be seen that the charge transfer resistance (R_CT_) has decreased over time with increase in biofilm formation.

### Iron Reduction by NDH-2 Expressing Cells

**FIGURE 10:**
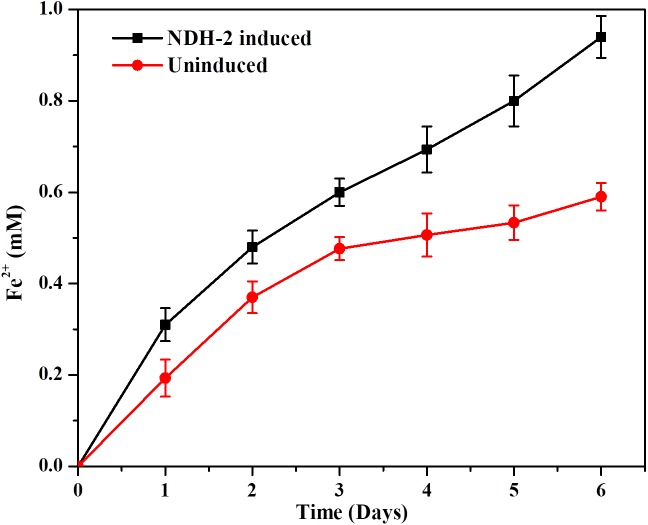
Iron reduction by NDH-2 and wild type cells using Ferrozine assay with respect to time.

The capacity of NDH-2 induced cells to reduce the soluble iron forms was determined by using ferric citrate as the only final electron acceptor. In order to prevent the aerobic reduction of ferric citrate or oxygen acting as final electron acceptor to *E. coli*, media was flushed with oxygen free N_2_-CO_2_ mixture. It was found that the expression of *ndh* gene in *E. coli* has enhanced its ability to reduce the Iron (III) citrate with lactate as carbon under anaerobic conditions. Readings (concentrations) from control sample which was blank media with iron (III) citrate and no culture was subtracted from the NDH-2 and wild type culture reading to rule out the chemical reduction of iron citrate. The maximum amount of ferric citrate reduced by induced culture was 0.94 mM compared to wild type culture which was 0.59 mM by the end of the 6th day which was ∼1.6 fold higher than the wild type (Figure 10). In addition to the total ferric citrate reduced by the end of day 6, it was also observed that the rate of reduction was same in both induced and wild type cultures till 3rd day while the induced culture almost continued at the same rates until day 6, wild type cultures showed reduced reduction rates after day 3. The enhanced reduction of ferric form to ferrous form can signify the enhanced extracellular electron transport as a result of NDH-2 expression.

**FIGURE 11:**
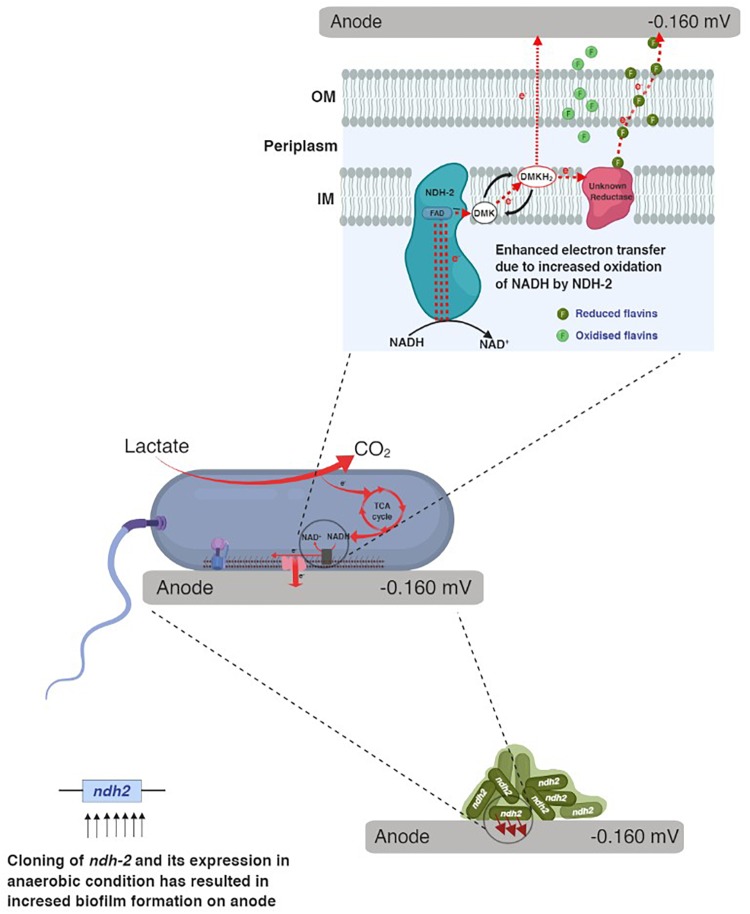
A hypothetical mechanism for extracellular electron transport in NDH-2 induced cells.

When carbon substrates like lactate were added to the reactor, *E. coli* utilizes carbon and produces electron donors like NADH, FADH_2_ which donates the electrons to the electron transport chain. In electron transport chain, primary dehydrogenases like NADH dehydrogenase oxidize the NADH and simultaneously reduce the quinones in the quinone pool. When *E. coli* was grown in bioelectrochemical system under anaerobic conditions with no electron acceptor, the electrons can only be delivered to the electrode (anode) via quinone pool or quinone oxidase either by mediated electron transport or by unknown direct electron transport route. Figure 11 shows the possible route for electron transport to the electrode in NDH-2 induced cells.

In the whole cascade of reactions, NADH and its oxidation is the limiting factor for the reduction of quinone pool (dimethyl menaquinone pool in case of NDH-2) and thus the overall electron flux. Thus in order to increase the electron flux, one of the possible options is to increase the NADH oxidation. NDH-2 being expressed only in the presence of excess oxygen, limits its application in the anaerobic conditions, so in order to control its activity even in the absence of oxygen and increase the NADH oxidation, NDH-2 gene has been cloned into the *E. coli*, where its expression can be controlled. The excessive electrons as a result of NADH oxidation by NDH-2 were found to entering the extracellular electron transport at quinone pool or at quinone oxidase. Immediate increase in current production after addition of riboflavin to the NDH-BES also suggests the same. In addition to this, enhancing the redox pool and electron flux of the cell can also be used for the synthesis of the high energy reduced products using bio-electrosynthesis systems. Further investigations have to be done for optimizing the reactions and metabolic circuits producing the reduced NAD molecules and in order to effectively utilize the carbon substrates to recover the redox pool.

## Conclusion

To efficiently increase the bioelectricity produced from a BES, we have increased the electron flux into the extracellular electron transport by cloning and expressing the NADH dehydrogenase II from *Bacillus subtilis* into the *Escherichia coli* which can increase the oxidation NADH produced in the central metabolism. This study has demonstrated the ability of NADH dehydrogenase II to enhance the metabolic flux and electron flux by increasing the NADH oxidation in the cell. Higher levels of total NAD pool specifically its oxidized form in the NDH-2 induced cells can be an indication of the increased electron transfer into the electron transport chain and increased catabolic activity in the cell. Subsequently, the *ndh* gene expression has led to a nearly nine-fold increase in current production from +0.52 to +4.7 μA and ∼1.6 fold increase in iron (III) reduction rates from 0.59 mM to 0.94 mM when compared to wild type culture, which shows that the *ndh* gene overexpression has led to a significant increase in extracellular electron transport. Further more, the activity of NDH-2 had a positive effect on biofilm formation on the anode which has significantly increased the extracellular electron transport due to direct electron transport. This study portrays the effect of NDH-2 activity on bioelectrogenesis and highlights the importance of NAD pool and activities of enzymes like NADH dehydrogenases on overall bioelectrogenesis.

## Author Contributions

SVM and KVK conceived the study and wrote the manuscript. KVK constructed the NDH-2 strain, purified the NDH-2 protein, and conducted the bioelectrochemical studies.

## Conflict of Interest Statement

The authors declare that the research was conducted in the absence of any commercial or financial relationships that could be construed as a potential conflict of interest.
