# Supplementary material for: Purification and Characterization of NDH-2 Protein and Elucidating Its Role in Extracellular Electron Transport and Bioelectrogenic Activity
Source: Front Microbiol. 2019 May 7;10:880. doi: 10.3389/fmicb.2019.00880 (PMC6513898; doi:10.3389/fmicb.2019.00880)
Supplement: Supplementary file 1 [file Table_1.DOCX]

**Purification and Characterization of NDH-2 Protein and Elucidating its Role in Extracellular Electron Transport and Bioelectrogenic Activity**

Vamshi Krishna K and S Venkata Mohan*

Bioengineering and Environmental Sciences Lab, CEEFF, CSIR- Indian Institute of Chemical Technology, Hyderabad 500 007, India

Email: vmohan_s@yahoo.com Telephone: 00-91-040-27191664

**Supplementary figure captions**

**SFig 1:** Blank or uninoculated control (A) Chronoamperometry (B) PEIS (C) Cyclic voltammetry










**SFig 1:** Blank or uninoculated control (A) Chronoamperometry (B) PEIS (C) Cyclic voltammetry
